# Microstructural Characterization of Friction Stir Welds of Aluminum 6082 Produced with Bobbin Tool

**DOI:** 10.3390/ma17194738

**Published:** 2024-09-27

**Authors:** Mateusz Kopyściański, Stanisław Dymek, Carter Hamilton, Aleksandra Węglowska, Izabela Kalemba-Rec

**Affiliations:** 1 Faculty of Metal Engineering and Industrial Computer Science, AGH University of Science and Technology, 30-059 Kraków, Poland; mateusz.kopyscianski@gmail.com (M.K.); gmdymek@cyfronet.pl (S.D.); kalemba@agh.edu.pl (I.K.-R.); 2 Department of Mechanical and Manufacturing Engineering, College of Engineering and Computing, Miami University, Oxford, OH 45056, USA; 3 Łukasiewicz—Upper Silesian Institute of Technology, The Welding Centre, 44-100 Gliwice, Poland; aleksandra.weglowska@is.lukasiewicz.gov.pl

**Keywords:** bobbin tool, friction stir welding, microstructure, simulation, aluminum

## Abstract

This study utilized a bobbin tool to friction stir weld aluminum 6082 workpieces under two sets of process parameters: a tool rotation speed of 280 rev/min with a weld velocity of 280 mm/min (280/280) and a tool rotation speed of 450 rev/min with a weld velocity of 450 mm/min (450/450). The weld microstructures were characterized through optical microscopy utilizing polarized light and through transmission electron microscopy (TEM) and scanning electron microscopy (SEM) coupled with chemical analysis by energy dispersive spectroscopy and electron back scatter diffraction. The microstructural studies were supplemented by hardness measurements (Vickers) performed on the same sections as the metallographic examinations. The produced weldments were free from cracks and any discontinuities. Fine, equiaxed grains that were several microns in size characterized the stir zones (SZs), and the advancing (AS) and retreating (RS) sides revealed distinct microstructural features. On the AS, the transition from the thermo-mechanically affected zone to the SZ was well defined and sharp, but on the RS, the transition appeared as a continuous, gradual change in microstructure. The lower weld energy (280/280) produced lower hardness in the stir zone than the higher energy weld (450/450), ~95 HV1 versus ~115 HV1; however, the 280/280 welds showed higher tensile strengths than the 450/450 welds, ~238 MPa as opposed to ~172 MPa. These behaviors in mechanical performance correlated with the temperature histories produced by each set of weld parameters in relation to the precipitation behavior of the alloy. The fracture characteristics of the weldments were notably different with the 450/450 sample fracturing in a quasi-brittle manner with slight plastic deformation and the 280/280 sample fracturing ductilely. A numerical simulation supported the investigation by elucidating the temperature and material flow behavior during the joining process.

## 1. Introduction

**Figure 1:**
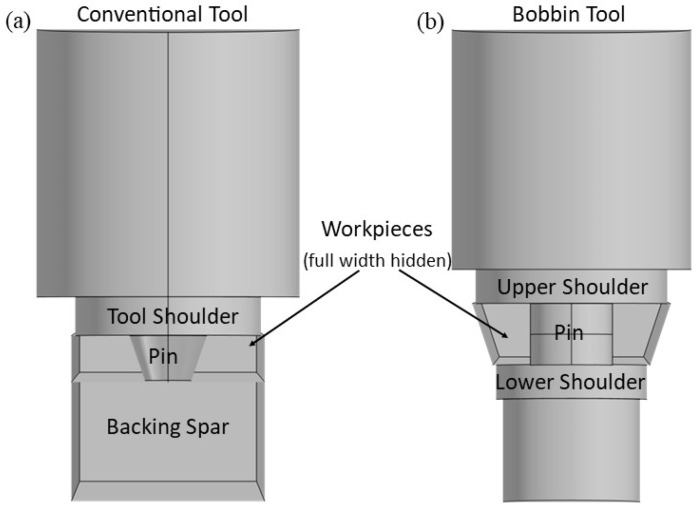
Schematic of the FSW process: (**a**) using a conventional tool and (**b**) using a bobbin tool.

Bobbin tool friction stir welding (BTFSW) is a variant of the conventional friction stir welding process. Friction stir welding (FSW) is now a mature technology that is frequently utilized to join metallic materials, especially precipitation-hardened aluminum alloys such as 2xxx, 6xxx, and 7xxx series aluminum alloys, which are considered difficult to weld by traditional fusion methods. The welding process takes place below the melting point or solidus temperature of the alloy and thus overcomes any detrimental effects that can arise from melting and re-solidification [1,2,3,4,5]. The most recent reviews regarding the FSW technology are presented in Refs. [6,7]. The concept of the bobbin tool is as old as FSW itself. The first description of this tool was written by Thomas et al. [8]. The fundamental concept of the bobbin tool is the introduction of a bottom shoulder that engages with the bottom surface of the workpiece. Thus, a bobbin tool consists of three parts: the upper shoulder, the pin (acting similarly to the standard FSW), and the lower shoulder [9,10,11]. Figure 1 shows the schematic differences between the FSW with a conventional tool (Figure 1a) and with a bobbin tool (Figure 1b).

The primary function of the lower shoulder is to support the material (replacing the backing spar/rigid backing), while the upper shoulder applies pressure to the workpieces. This pressure, however, is much smaller compared to the traditional FSW process, and consequently, the amount of heat introduced by friction is less than in the conventional FSW process. The pin, like in a conventional tool, mixes the material and supplies additional heat to the process. The diameter of the pin should be large enough to withstand the forces exerted by the welded material, yet small enough to mix the workpieces. In addition, the distance between the two shoulders should be slightly smaller (~1 mm) than the thickness of the workpieces to ensure a vertical friction force during processing and to contain the material within the process zone [11,12,13]. The great advantage of welding with a bobbin tool is that it eliminates the need of backing support, the use of which in some cases is difficult or even impossible. A unique benefit of this technique, therefore, is the ability to weld closed sections without using this rigid backing. Additionally, the use of a bobbin tool alleviates weld defects on the weld ridge side and ensures more uniform heat generation during the process. In contrast to the conventional FSW, bobbin tool friction stir welding (BTFSW) allows for the joining of materials oriented not just horizontally. Further, depending on the bobbin tool design, the pin and shoulders can rotate at different speeds and even in opposite directions. This allows for better optimization of the welding conditions, e.g., by setting a high rotation speed of the pin for mixing and a relatively lower rotation speed of the shoulders for heat input. In a typical BTFSW weld, the same microstructural zones are observed as in the conventional FSW, i.e., the stir zone (SZ), the thermo-mechanically affected zone (TMAZ), and the heat-affected zone (HAZ) [11,12,13].

The 6082 alloy belongs to the group of precipitation-strengthened alloys, and it is widely applied in the industry due to its resistance to stress corrosion cracking and to its good corrosion resistance in general. This aluminum alloy is considered as a material candidate for applications in shipbuilding, rail stock, automotive, and many other industries [7]. It has been already demonstrated that the FSW welds of this alloy exhibit good fatigue resistance that broaden its potential applications [7,8]. Investigations of welds of aluminum alloys produced with a bobbin tool are only sporadically reported in the world literature. Threadgill et al. [14], Sued- et al. [15], and the work of Esmaily et al. [16] compare the microstructure of 6005-T6 welds made with a conventional tool and a bobbin tool. Some research on the BTFSW of the 6082 aluminum alloy was presented by Wan et al. [17,18].

The goal of this proposed research, therefore, was to analyze the microstructure and mechanical properties of aluminum 6082 joints fabricated with the BTFSW method. Two unique combinations of rotation and welding speeds were considered: a tool rotation speed of 280 rev/min with a weld velocity of 280 mm/min and of 450 rev/min with a weld velocity of 450 mm/min. Fine, equiaxed grains characterized the stir zones of each joining condition with hardness recovery occurring in relation to the TMAZ. The hardness of the stir zone for the 450/450 condition was higher than that of 280/280 and even exceeded the baseline hardness of the alloy. Such behaviors are correlated to the microstructural, precipitation, and temperature characteristics during BTFSW. Ultimately, for either set of process parameters, this study demonstrates the efficacy of BTFSW to join 6082 workpieces, thus more confidently availing the method to industry.

## 2. Materials and Methods

**Figure 2:**
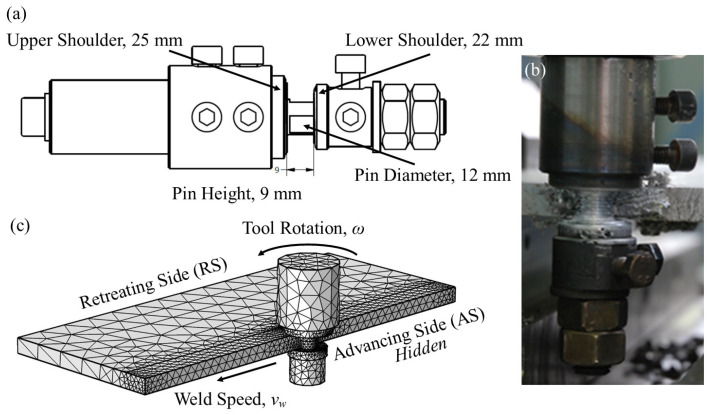
(**a**) Schematic of bobbin tool with dimensions, (**b**) actual bobbin tool during friction stir welding, and (**c**) numerical simulation cross-section showing bobbin tool with workpieces.

Bobbin tool friction stir welding of 10 mm thick plates of a 6082-T6 commercial aluminum alloy was performed at two different tool rotation speeds and weld velocities: a tool rotation speed of 280 rev/min with a weld velocity of 280 mm/min and of 450 rev/min with a weld velocity of 450 mm/min. The concentrations of the main alloying elements (determined by the Perkin Elmer OP-TIMA 7300 DV ICP Optical Emission Spectrometer, PerkinElmer, Waltham, MA, USA) of the base alloy are presented in Table 1. A schematic of the high-speed steel bobbin tool utilized in this investigation is presented in Figure 2a with the actual tool shown during welding in Figure 2b. The tool has an upper shoulder diameter of 25 mm, a lower shoulder diameter of 22 mm, a pin diameter of 12 mm, and a pin length of 9 mm. The investigation was supported by a numerical simulation to highlight temperature and material flow during the joining process. The development, details, and experimental validation of this simulation are thoroughly presented in reference [19], and a representative cross-section of the solid model from the simulation is presented in Figure 2c, which shows the bobbin tool and workpieces. The simulation in Comsol couples the “Heat Transfer in Solids” and “Non-Isothermal Flow” studies to obtain the temperature distributions during welding. The mesh comprises 25,433 tetrahedral, 7128 triangular, 936 edge, and 68 vertex elements. In the area around the tool/workpiece interfaces and within the flow capable region, a fine mesh was applied that ranged in size from 1.19 mm near the tool center to 6.29 mm near the inlet and outlet of the flow region. A mesh study demonstrated that a finer mesh did not substantially change the simulation results.

Transmission electron microscopy (JEOL-200CX TEM microscope, JEOL Ltd., 3-1-2 Musashino, Akishima, Tokyo 196-8558, Japan) was employed to assess the precipitation characteristics of the baseline material. Thin foils were prepared from the material by excising 3 mm disks and then electropolishing them in a solution of HNO_3_ and CH_3_OH (1:2) at −30 °C and 12 V. The microstructures of the welds were examined by optical microscopy utilizing polarized light and by scanning electron microscopy (SEM) with electron back scatter diffraction capabilities (EBSD) (FEI, Labsoft, ul. Pulawska 469 02-844 Warsaw, Poland). All of the examinations were carried out on sections perpendicular to the welding direction along the mid-plane of the workpieces’ thickness. The microstructural studies were supplemented by the hardness measurements (Vickers) on the same sections as the metallographic samples. Hardness profiles on a weld cross-section along the line of the mid-thickness plane were constructed from these measurements. The applied load was 1 kg, and the distance among the testing points was 1.0 mm. Tensile tests were performed for the mechanical characterization of the welds. During the welding process, temperature measurements were also performed using a Vigocam v50 thermal imaging camera (VIGO Photonics, Poznańska 129/133, 05-850 Ożarów Mazowiecki, Poland).

## 3. Results and Discussion

**Figure 3:**
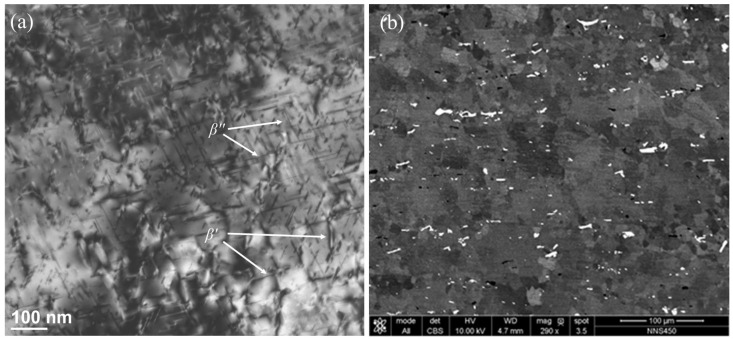
Microstructure of the base material with precipitates of strengthening phases: (**a**) TEM and (**b**) SEM.

The TEM microstructure of the base material in the initial state (Figure 3a) reveals numerous precipitates of the metastable *β*″ phase in the form of needles and the *β*′ phase in the form of rods. The SEM microstructure reveals second-phase distributions in the base material after extrusion (Figure 3b).

**Figure 4:**
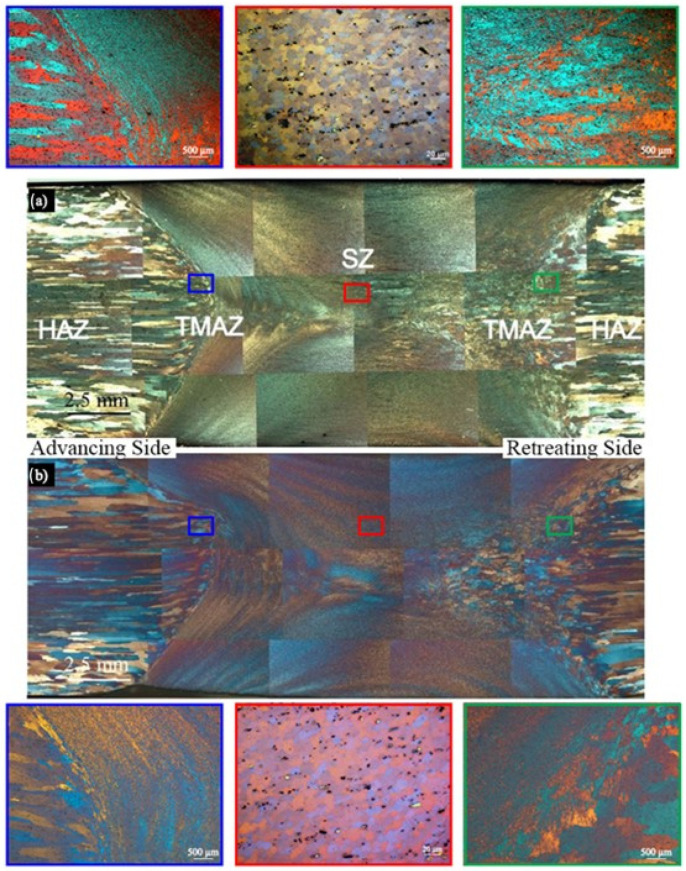
The structure of weldments in macroscale: (**a**) 280 rev/min and (**b**) 450 rev/min.

The cross-sections of the weld microstructures (in macro scale) from the BTFSW joints are shown in Figure 4 for both of the tool rotation speeds, i.e., at 280 rev/min and at 450 rev/min. An important difference between the weld microstructures produced during conventional FSW and BTFSW is the shape of the stir zone itself. The use of two shoulders produces a weld with a shape that resembles an “hourglass” as shown in the figure. The weld width measured at the mid-thickness of the cross-section for 280 rev/min is 13.95 mm, and that for 450 rev/min is 14.54 mm. The examined joints exhibit three characteristic areas, i.e., the heat-affected zone (HAZ), the thermo-mechanically affected zone (TMAZ), and the stir zone (SZ), as indicated in Figure 4.

The analysis of the weld zones at the tool rotation speeds of 280 and 450 rev/min did not reveal significant differences in the grain size across the three characteristic areas. However, a detailed examination of the stir zone showed that at 280 rev/min (Figure 4a), there was a slightly smaller grain size compared to the stir zone at 450 rev/min (Figure 4b) (though the differences are within the limits of measurement error). This phenomenon can be explained by the fact that at a higher tool rotational speed (450 rev/min), a higher welding temperature is observed, promoting slightly more grain growth than at the lower speed (280 rev/min) and yielding the minor differences in the microstructure of the stir zones.

**Figure 5:**
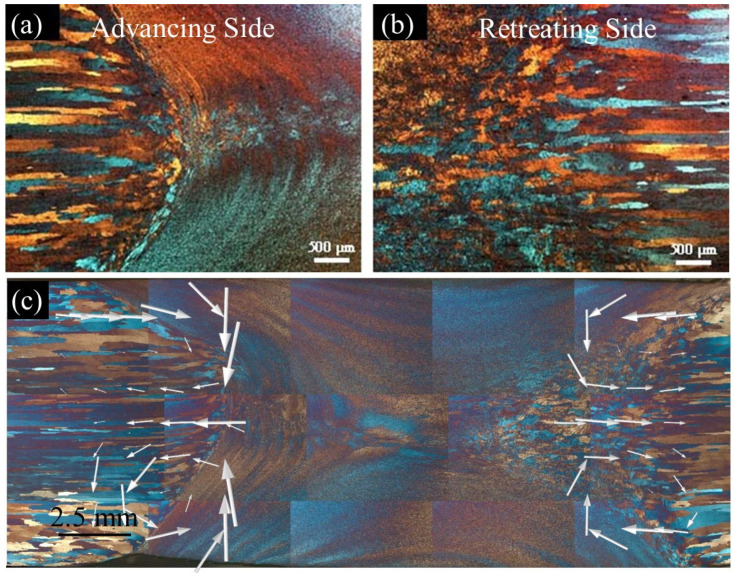
Microstructure of a thermo-mechanical affected zone for 450 rev/min: (**a**) the advancing side, (**b**) the retreating side, and (**c**) the material flow map.

As further highlighted in Figure 5a, on the advancing side, the interface between the stir zone and the base material is quite distinct, but on the retreating side, it is relatively diffuse and not as clearly defined as shown in Figure 5b. These boundary patterns are consistent with previous studies on conventional welds, which demonstrate that on the advancing side, the material flow at the interface contains a strong vertical component that produces the sharp boundary, while on the retreating side, the flow tends to contain a stronger horizontal component, creating the diffuse boundary as presented in Ref. [20]. Figure 5c overlays a material flow map from the simulation on the weld cross-section, with arrows indicating the flow direction and the arrow size indicating the relative magnitude. The image demonstrates the stronger vertical flow on the advancing side near the pin, and the stronger horizontal flow in this region on the retreating side, thus contributing to the unique stir zone/base material interfaces on each side of the weld.

Additionally, the structure of the SZ exhibits the characteristic pattern of bands referred to as “onion rings”, which are also observed in the conventional FSW welds. One mechanism for the formation of “onion rings” in precipitation-hardened aluminum alloys is the uneven distribution of the precipitates of intermetallic phases [21]. According to this theory, the rings are formed by alternating alloy layers that experienced higher and lower temperatures during the welding process depending on their original position within the cross-section relative to the tool (heat input source). The bands of the material with a higher-temperature thermal history are “richer” in the precipitates after cooling due to a greater extent of elements in solid solution during processing, i.e., reprecipitation upon cooling. In turn, the bands with a lower-temperature thermal history are “poorer” in precipitates due to the dissolution and/or ripening without reprecipitation upon cooling. This disparity in the precipitation creates a contrast in the microscopic images as these “onion ring” formations [21].

**Figure 6:**
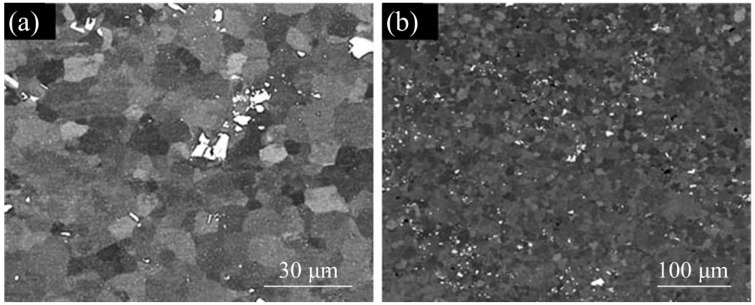
SEM microstructure of the stir zone: (**a**) equiaxial grains in the stir zone and (**b**) uniform distribution of intermetallic phases (light particles).

In the stir zone, fine, equiaxed grains are visible, as seen in Figure 6a. The reason for the formation of a fine-grained microstructure is that the center of the weld (stir zone) is subjected to significant plastic deformation combined with the physical flow of material (mixing) and a simultaneous significant temperature increase. The temperature measured during the process (just behind the tool) was about 460 °C for 450 rev/min and approximately 430 °C for 280 rev/min. These temperatures are sufficient for dynamic recovery and dynamic recrystallization in aluminum alloys. Dynamic recrystallization in this zone forms a microstructure composed of fine equiaxed grains. The SEM observations (Figure 6b) from the backscattered electron detector, in addition to the information on the size and shape of the grains in the stir zone, also provided information about the distribution of the intermetallic phase inclusions (light particles). The bright contrast indicates the presence of elements with a higher atomic number. The EDS analysis of such particles carried out on the SEM revealed a greater manganese content. Comparing Figure 3b (second phase distributions in base material) and Figure 6b, it can be observed that the intermetallic phases were also fragmented and physically mixed in the stir zone.

**Figure 7:**
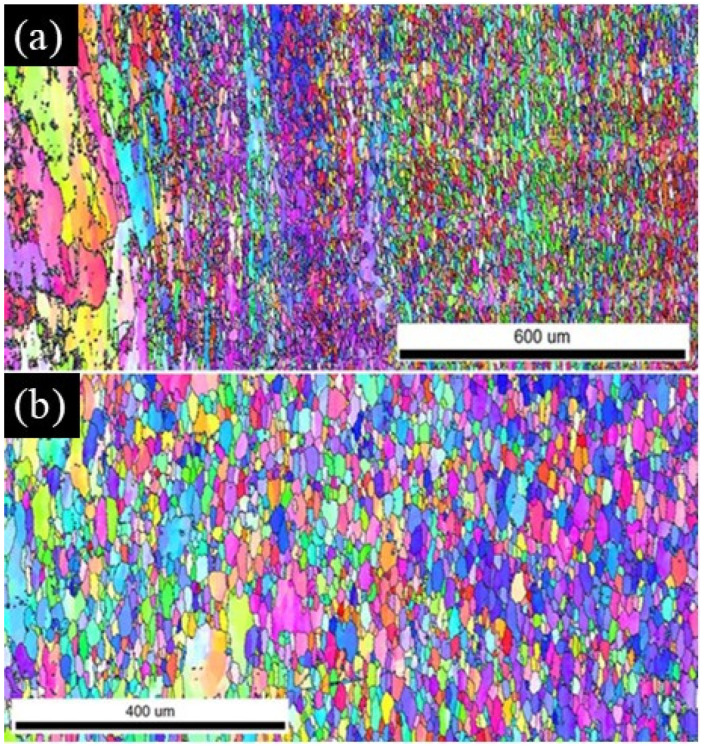
Exemplary EBSD images showing the shape and size of the grains for 450 rev/min: (**a**) on the advancing side and (**b**) in the stir zone.

**Figure 8:**
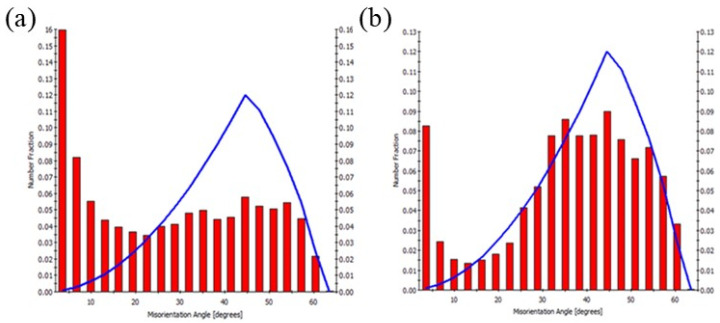
Histograms of grain boundary misorientations for the 450 rev/min sample: (**a**) in the area between the SZ and TMAZ and (**b**) in the stir zone.

The images of the microstructure obtained by the EBSD method (Figure 7) also provided information about the size and shape of the grains in the individual zones and the distribution of the misorientation angles between neighboring grains. The stir zone is characterized by small equiaxed grains with an average diameter of several microns. In the thermo-mechanical affected zone, the grains are larger and elongated due to the degree of deformation in this zone, which is less than in the SZ and at a lower temperature. The elongated shape of the grain indicates that dynamic recrystallization is incomplete. This phenomenon is confirmed by the misorientation distribution among the grains in the area between the SZ and TMAZ zones (on the advancing side) as shown in Figure 8 for 450 rev/min. Misorientation histograms are also superimposed on the McKenzie plot, which determines the theoretical distribution of the grain boundaries in polycrystalline with a regular structure and a random grain orientation. The experimental distribution of the misorientation disorders significantly differs from the course of this curve. In the analyzed area, there is a proportion of grains with disorientation angles below 15°, and therefore, there is also a small grain angle. In contrast, in the middle of the weld zone, the misorientations of the grain boundaries are more similar to the shape of the McKenzie line (more borders with misorientations in the range of 20°–60°), which may suggest that dynamic recrystallization took place in this area during the welding process (Figure 8b).

**Figure 9:**
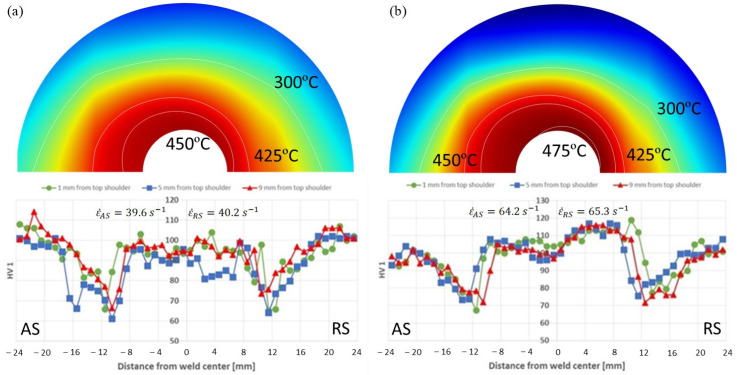
Hardness profiles for AS-welded samples: (**a**) 280 rev/min and (**b**) 450 rev/min. Correlating temperature maps from the simulation are shown above each plot.

The results of the hardness measurements in Figure 9 are presented as profiles reflecting the hardness changes in the cross-section of the weld. These profiles are in the shape of the letter “W”, which is typical for precipitation-hardened aluminum alloys [2]. The investigation revealed differences in the hardness profiles for welds combined with low welding energies (280/280 parameters) and higher energies (450/450 parameters). For the tested process parameters, the hardness decreased from both sides of the base material and reached the local minimum in the heat affected zone, but then increased in the middle of the weld. For the 280/280 sample in the stir zone, the hardness increased to about 95 HV1, but it was still less than the hardness of the base material, which was 105 HV1. In contrast, the hardness in the stir zone of the 450/450 sample increased to 115 HV1 and was, therefore, higher than for the base material. It is also noteworthy that the 280/280 sample displayed lower local minimums than the 450/450 sample. In addition, the distance between the local minima was higher in the sample with the 450/450 parameters.

The differences in the hardness profiles resulted from the temperature differences between the welding parameter sets in relation to the precipitation/dissolution behavior of the aluminum alloy, as well as the dynamic recrystallization behavior. Included in Figure 9 above, the hardness profiles are the predicted temperature distributions from the simulation on the plane 5 mm from the tool shoulder for each parameter set. The average strain rates on the mid-thickness reference line (6 mm behind the tool) extending from the TMAZ/SZ boundary to the weld center are also presented for the AS and RS. Due to the resulting heat input and temperatures, the dissolution and ripening of the strengthening phases in the alloy can contribute to the decrease in hardness within the thermo-mechanically affected zones. In this region, the grains were also larger and longer than in the SZ, indicating that the temperature was insufficient for dynamic recrystallization to occur. The temperatures in the heat-affected zone and the lack of plastic deformation from the tool causes ripening of the strengthening phases; thus, the decrease in the hardness was largest in this zone. The large differences in the hardness in the stir zones of both samples were caused by the higher temperatures in the 450/450 samples compared to the 280/280 samples, approximately 460 °C versus 430 °C, and by the higher strain rates promoting recrystallization. At these higher temperatures, the precipitates dissolved to a greater extent, and therefore, the effect of post-weld natural aging in the 450/450 sample was stronger. The influence of natural aging on hardness in the stir zone has been described in detail in [22,23] for aluminum alloys of the 7000 series.

**Figure 10:**
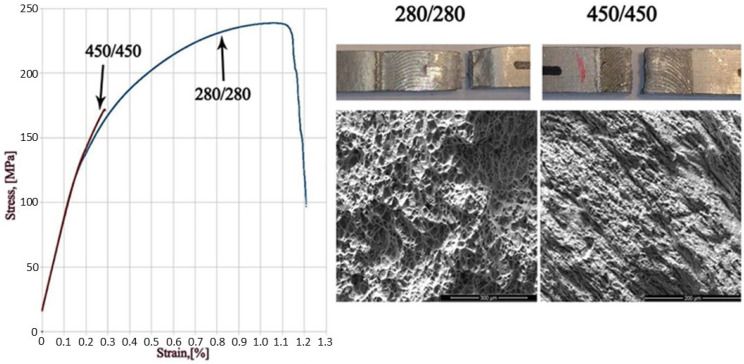
Tensile curves with fracture surfaces of the examined samples.

Tensile tests were also carried out on the welded panels. The tensile strengths of the welded samples were less than that of the base material, as summarized in Table 2. For the 280/280 parameter, the tensile strength was 238 MPa, and for the 450/450 sample, the tensile strength was 172 MPa. The large difference in the tensile strength of the tested samples is reflected in the nature of the fracture in both variants, as highlighted in Figure 10. For the lower rotational speed, the fracture was of a ductile nature, and the breakthrough of the sample took place in the heat-affected zone on the retreating side. For the higher rotation speed, the breakthrough occurred in the middle of the weld, and the fracture was quasi-brittle in nature. The physical reason for the brittleness of the 450/450 sample is associated with the higher processing temperatures. The temperature rise during the FSW promotes complex changes in the morphology of the strengthening phases, *β*″ and *β*′. The precipitates in the stir zone are partially or completely dissolved, and then, upon cooling, they reprecipitate and are subjected to ripening. With this reprecipitation, new secondary phases appear on grain boundaries and contribute to the brittle behavior. The stir zone hardness of the 450/450 sample is higher than for the 280/280 sample (Figure 9), indicating that a lower ductility in 450/450 sample would be expected.

## 4. Conclusions

This investigation has demonstrated that the FSW method with the use of a bobbin tool is an effective method of joining workpieces made of 6082 alloy. The use of such a tool eliminates the need for support under the joined pieces, and all of the tested joints were free from defects and welding imperfections. The stir zone (center of the weld) was characterized by very small and equiaxed grains with an approximate average diameter of several microns. The microstructure of the joint was unique on the advancing and retreating sides. The boundary between the thermo-mechanical zone and the stir zone on the advancing side was sharp and distinct, while on the retreating side, a continuous change in microstructure was observed in the transition between these zones. The use of a bobbin tool creates an hourglass weld which is wider at the top and tapered at the bottom. There were clear differences between the hardness profiles for lower and higher speeds of the welding tool: for a lower speed, the hardness profile showed lower hardness values in the stir zone, while for a higher speed, the hardness value in the stir zone was higher than the hardness of the parent material. A prominent difference was also observed in the mechanical behavior: the 450/450 sample fractured in a quasi-brittle manner with slight plastic deformation, while the 280/280 sample fractured ductilely. The yield and tensile strengths of the welded samples were lower than those of the parent material.

## Figures and Tables

**Table 1:** Main element contents in the examined alloy, aluminum 6082 (wt%).

Mg	Si	Mn	Fe	Cr	Zn	Cu	Ti
1.10	0.80	0.60	0.50	0.25	0.20	0.10	0.10

**Table 2:** Mechanical properties of tested samples.

Sample	Tensile Strength (MPa)	Yield Strength (MPa)	Elongation (%)
280/280	238	195	5.4
450/450	172	--	1.4
Base Material	295	250	8.0

## Data Availability

The raw data supporting the conclusions of this article will be made available by the authors on request.
